# Early identification of risk factors for obstructive sleep apnea hypopnea syndrome based on large language models

**DOI:** 10.3389/fmed.2026.1772777

**Published:** 2026-06-15

**Authors:** Lei Cheng, Juan Bai, Aizhu Liu

**Affiliations:** Department of Otolaryngology Head and Neck Surgery, Capital Medical University Affiliated Beijing Shijitan Hospital, Beijing, China

**Keywords:** chronic disease management, disease risk factor identification, family medicine and primary care, GPT-5.1, OSAHS

## Abstract

**Background:**

Obstructive sleep apnea hypopnea syndrome (OSAHS) is a highly prevalent sleep-related breathing disorder, yet it is frequently missed in its early stages. Early identification of OSAHS-related risk factors and early signals from real-world patient-generated text may enable timely screening and intervention. However, existing approaches primarily rely on structured clinical data or end-to-end classification models, which are limited in handling unstructured, colloquial descriptions and highly imbalanced class distributions.

**Objectives:**

This study aimed to develop and evaluate a large language model-based framework for early identification of OSAHS-related risk factors and early signals from free-text descriptions, enabling robust text-level risk stratification with improved interpretability.

**Methods:**

We proposed OSAHSrisk-LLM, a large language model-based framework that analyzes patient-generated text using a relevance-aware and ontology-constrained reasoning strategy. The framework first determines whether a text contains OSAHS-related risk information, and then applies stepwise, evidence-based reasoning to identify early signals and risk factors under predefined clinical knowledge guidance. Linguistic variability in real-world narratives is addressed by normalizing extracted concepts into standardized clinical terms. We evaluated the framework on a corpus of real-world Chinese text describing sleep-related experiences and compared it with multiple baseline text classification models.

**Results:**

OSAHSrisk-LLM achieved an overall accuracy of 92.9% in the four-class text-level classification task, numerically outperforming the baseline models including CNN, Text-CNN, Transformer, and BERT on the current dataset. Notably, the proposed framework demonstrated strong robustness under highly imbalanced class distributions and improved identification of minority categories.

**Conclusion:**

Our findings suggest that large language models, when integrated with clinical knowledge constraints and structured reasoning strategies, can effectively extract early OSAHS-related risk information from patient narratives. The proposed framework demonstrates the potential of large language models to identify textual mentions of OSAHS-related risk factors and early signals in patient-generated narratives. Further validation against clinically confirmed OSAHS diagnoses and prospective screening cohorts is required before use in real-world clinical risk screening.

## 1 Introduction

OSAHS is a common sleep-related breathing disorder characterized by recurrent episodes of upper airway obstruction during sleep, resulting in intermittent hypoxia and sleep fragmentation (1, 2). OSAHS has been associated with a wide range of adverse health outcomes, including cardiovascular disease, metabolic disorders, cognitive impairment, and reduced quality of life. Despite its high prevalence, OSAHS remains substantially underdiagnosed, particularly in its early stages. Early symptoms are often subtle, intermittent, or nonspecific, and many individuals do not seek medical attention until the condition has progressed (3, 4). As a result, opportunities for early screening and timely intervention are frequently missed.

Early identification of OSAHS risk factors and early signals is essential for improving screening efficiency and reducing the burden of undiagnosed disease. In clinical practice, risk assessment typically relies on structured information such as demographic characteristics, anthropometric measurements, comorbid conditions, and standardized questionnaires (5, 6). However, in real-world settings, individuals often describe sleep-related experiences, lifestyle habits, and bodily changes in informal and unstructured ways (7, 8). Patient-generated text, including self-reported narratives and preclinical descriptions, may contain valuable information related to OSAHS risk factors and early signals (9, 10). Extracting such information is challenging due to the colloquial nature of language, high variability in expression, and the presence of subjective or incomplete descriptions.

Existing computational approaches for analyzing sleep-related text data largely depend on rule-based systems or end-to-end classification models (11, 12). Rule-based methods require extensive manual effort and lack scalability, while end-to-end models often struggle to generalize in the presence of highly imbalanced class distributions (13, 14). Moreover, these models typically provide limited interpretability, making it difficult to understand how specific risk factors or early signals contribute to the final prediction. Such limitations restrict their applicability in clinical screening scenarios, where transparency and reliability are critical (15, 16).

Recent advances in large language models have demonstrated strong capabilities in understanding natural language and extracting structured information from unstructured text (17, 18). These models offer new opportunities for identifying clinically relevant information from patient narratives (19). However, directly applying large language models as black box classifiers may introduce risks such as unreliable outputs and limited control over the inference process. For clinical applications, especially those involving early risk screening, it is important to guide model reasoning using domain knowledge and to ensure that extracted information is grounded in the original text (20).

Recent studies have explored the extraction of disease-related risk factors from patient-generated text using natural language processing and large language models. Gu et al. conducted a mixed-method study on Zhihu comment data to identify risk factors for allergic rhinitis using a topic-enhanced word embedding model (21). Their approach combined topic modeling and clustering analysis to reveal population-level risk patterns from online discussions. While effective for uncovering latent themes, this method relies on statistical topic distributions and does not provide fine-grained, text-level evidence attribution. More recently, Mao et al. proposed an LLM-based framework to identify kidney stone risk factors from patient experience narratives (22). Their study demonstrated the feasibility of large language models in extracting structured risk information from unstructured health text without task-specific model training. However, the extraction process mainly follows an end-to-end inference paradigm without explicit semantic constraints or relevance screening mechanisms.

Compared with these studies, our work introduces a knowledge-guided, ontology-constrained framework specifically designed for early OSAHS risk screening, OSAHSrisk-LLM. First, we define a domain-specific ontology covering both risk factors and early signals (Supplementary Tables S1, S2), ensuring that all extracted information is clinically interpretable and semantically standardized. Second, we incorporate a relevance-aware gating stage to filter non-informative texts before inference, reducing noise from general sleep complaints. Third, we employ a stepwise reasoning strategy that prioritizes early signal identification, followed by risk factor extraction, with mandatory evidence-span attribution for each extracted concept.

Our main contributions are as follows:

1. We develop a relevance-aware and ontology-constrained LLM framework for early identification of OSAHS-related risk factors and early signals from free-text patient narratives, addressing the challenges posed by linguistic variability and highly imbalanced data distributions.
2. We introduce a structured, stepwise reasoning strategy that prioritizes the identification of early signals before risk factors and enforces evidence attribution and concept normalization, enabling transparent and clinically interpretable text-level risk stratification.
3. We conduct an empirical evaluation on real-world patient-generated text, demonstrating that the proposed framework achieves superior and more stable performance compared with multiple baseline models, while maintaining improved recognition of minority risk categories.

The structure of the remainder of the paper is as follows: Section 2 provides a detailed description of the materials and methods, Section 3 outlines the experiments, Section 4 discusses the results and provides the discussion, and finally, Section 5 concludes the paper.

## 2 Methods

### 2.1 Framework

**Figure 1:**
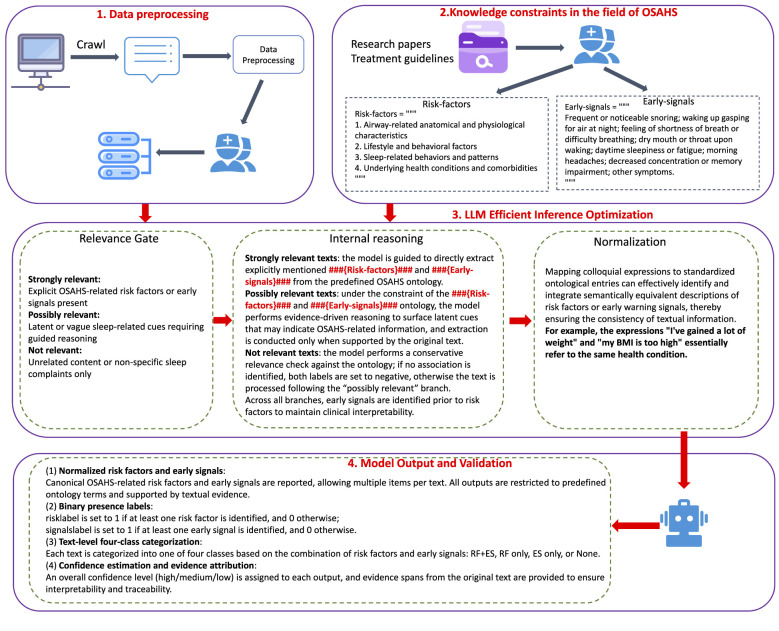
A framework for early identification of OSAHS risk factors and early signals based on large language models. (1) Data collection and preprocessing, aiming to obtain a high-quality corpus of patient-generated text describing sleep-related experiences. (2) Construction of OSAHS clinical knowledge, in which risk factors and early signals are summarized and organized based on relevant literature and clinical expertise to guide model reasoning. (3) Structured LLM inference with relevance gating, ontology constraints, and evidence attribution, where texts are first screened for relevance to OSAHS-related risk, followed by ontology-guided and evidence-based reasoning to identify early signals and risk factors, with normalization of extracted concepts to standardized clinical terms. (4) Structured output and text-level categorization, producing normalized risk factors and early signals with evidence attribution, and generating final text-level risk labels for downstream evaluation and analysis.

The framework used in this study is composed of four parts, as illustrated in Figure 1. The framework proposed in this study, termed OSAHSrisk-LLM, is illustrated in the final framework diagram and consists of four main components. The first component involves data preprocessing, where patient-generated free-text sleep narratives are collected and normalized to support subsequent analysis. The second component incorporates domain knowledge constraints, in which clinical expertise and established medical knowledge related to OSAHS are embedded to guide model reasoning and improve reliability. The third component is structured model inference, enabling the large language model to perform stepwise reasoning that integrates relevance assessment and clinically informed decision-making. The final component focuses on output generation with validation, ensuring that the extracted OSAHS risk factors and early signals are evidence-based and clinically interpretable. Through this multi-stage design, the framework is able to effectively analyze unstructured and colloquial sleep-related narratives for early OSAHS risk identification.

This study adopted a deterministic stepwise inference protocol to systematically guide the reasoning process of GPT-5.1. All analyses were performed using the GPT-5.1 interface under the default inference configuration provided by the platform at the time of experimentation. The same structured prompting strategy, reasoning workflow, ontology constraints, and postprocessing procedures were applied consistently to all text samples. The protocol achieves adaptation to public health screening scenarios and reasoning quality control through a four-stage progressive workflow. The first stage is role and scope definition: the model's functional positioning was clarified as a “public health screening assistant” via system instructions, while strictly defining the model's boundaries—explicitly prohibiting the generation of any form of medical diagnosis conclusions or treatment recommendations to avoid overstepping outputs beyond its tool attributes. The second stage is the relevance gate: a three-level classification system was established to assess the relevance of input texts, with specific criteria as follows: texts that explicitly mention any predefined ontology concept are categorized as “strongly relevant”; those containing only implicit or vague sleep-related cues (without corresponding explicit ontology concepts) are classified as “possibly relevant”; and texts with no sleep-related information are deemed “not relevant.” The third stage is Structured reasoning execution: for the screened relevant texts (strongly relevant and possibly relevant), the model completes three core reasoning tasks in a fixed sequence: (1) early signal extraction, i.e., identifying initial clues related to the public health screening objectives in the texts; (2) risk factor extraction, further screening and refining key variables that may affect screening results; (3) ontology constraint verification, ensuring that the extracted content complies with the semantic norms and logical requirements of the predefined ontology system. The fourth stage is output validation: a three-fold verification standard was established to control the quality of all extracted results, requiring each extracted item to meet the following criteria: (1) accurate mapping to entries in the predefined ontology library; (2) support by explicit textual evidence without subjective speculative content; (3) simultaneous return of evidence spans corresponding to the evidence, ensuring the traceability and verifiability of the results.

### 2.2 Dataset

The dataset used in this study was collected from two widely used Chinese online health platforms: Xunyiwenyao (www.xywy.com) and 39 Health (www.39.net) (23). These platforms allow users to consult physicians and share health related concerns through free text descriptions, providing a large volume of real-world patient-generated narratives (24). The collected texts primarily describe sleep-related experiences, lifestyle habits, physical conditions, and subjective symptoms, reflecting how individuals naturally express health concerns outside formal clinical settings.

All texts were publicly accessible and anonymized prior to analysis. No personal identifiers were collected or used in this study. The dataset represents a real-world corpus of colloquial Chinese health related text, characterized by informal language, diverse expression styles, and varying levels of detail. Such characteristics make the dataset suitable for evaluating the ability of large language model based methods to identify OSAHS related risk factors and early signals from unstructured patient narratives (25). Publicly available patient-generated texts were collected from the online health consultation platforms Xunyiwenyao and 39 Health over a 5-year period (2021–2025). Data collection was conducted using OSAHS-related Chinese search terms associated with sleep symptoms, snoring, breathing problems, daytime sleepiness, and related health concerns.

In total, 963 text samples were initially collected. During preprocessing, 156 duplicate or near-duplicate entries were removed, along with 74 advertisements or promotional texts, 53 texts irrelevant to sleep or health topics, and 120 texts containing insufficient information for annotation and analysis. The final dataset consisted of 560 texts, including 247 from Xunyiwenyao and 313 from 39 Health. These samples served as the raw corpus for subsequent preprocessing and analysis. This study was conducted as a public health-oriented infodemiology investigation. The aim was to identify population-level early risk patterns of OSAHS from naturally occurring, user-generated health narratives, rather than to diagnose or predict disease status at the individual level. All data were collected exclusively from publicly accessible online health platforms (Xunyiwenyao and 39 Health), where users voluntarily share information without access restrictions. No private, login-protected, or paid content was accessed. This data collection strategy is consistent with prior infodemiology and digital epidemiology studies that utilize public online data without requiring formal ethical approval. To further protect privacy, we implemented strict safeguards, including manual screening, removal of any residual identifiers, and a strict policy prohibiting re-identification attempts.

### 2.3 Data preprocessing

The raw text data used in this study were collected from two major Chinese online health platforms, Xunyiwenyao and 39 Health, where users describe health concerns and consult physicians through free-text narratives. Similar to social media data, such patient-generated text exhibits a high degree of ambiguity and linguistic variability. The descriptions are often informal and colloquial, and may contain incomplete sentences, vague references, emotional expressions, or implicit health information. These characteristics pose substantial challenges for traditional text processing pipelines, which typically require extensive manual preprocessing, rule design, and feature engineering. In practice, ambiguous or weakly expressed texts are often filtered out, which may lead to the loss of potentially valuable early risk information.

Large language models, such as GPT-5.1, have demonstrated strong capabilities in contextual understanding and semantic reasoning due to pretraining on large-scale and diverse corpora. These models can tolerate colloquial expressions, spelling inconsistencies, and unclear descriptions, and can infer latent meanings from surrounding context. Such properties make large language models particularly suitable for analyzing real-world patient narratives that resemble social media text. Motivated by these advantages, we adopted a large language model-based framework to process and analyze the collected text, allowing ambiguous and implicit information to be retained and interpreted rather than aggressively filtered during preprocessing.

To balance data quality and authenticity, we applied a conservative preprocessing strategy. First, duplicate and near-duplicate entries were removed. Second, texts containing advertisements, irrelevant content, or no meaningful health-related information were excluded. Third, entries clearly unrelated to sleep or health conditions were filtered through manual inspection supported by keyword screening (26). Importantly, no aggressive linguistic normalization was performed. Colloquial expressions, repetitions, subjective descriptions, and emotionally charged language were intentionally preserved to reflect real-world patient expression and to evaluate the robustness of the proposed framework under realistic conditions.

After preprocessing, 560 text samples were retained from the initial 963 collected texts. All retained samples were independently annotated by two clinicians with experience in sleep-related disorders following a predefined annotation manual. The annotators labeled OSAHS-related risk factors and early signals according to the ontology definitions provided in Supplementary Tables S1, S2, based on established clinical knowledge and relevant medical literature.

Only information explicitly stated or strongly implied in the text was annotated, while hypothetical, speculative, irrelevant, or transient acute conditions were excluded. For texts containing vague or ambiguous expressions, the annotators adopted a conservative strategy and deferred labeling until further discussion.

After independent annotation, disagreements were resolved through structured consensus review and discussion until mutual agreement was reached. The final consensus annotations were used as the reference standard for evaluating the proposed framework and baseline models. This consensus-based protocol was designed to improve labeling consistency and clinical relevance.

### 2.4 Knowledge constraints in the field of OSAHS

To guide model reasoning and ensure clinical consistency, we defined an OSAHS specific ontology covering two target categories: risk factors and early signals. Risk factors were organized into clinically meaningful groups, including anatomical and physiological characteristics, lifestyle related factors, sleep-related behaviors, and comorbid health conditions. Early signals were defined as patient perceived or observer reported manifestations associated with sleep disordered breathing, such as nocturnal symptoms, morning discomforts, and daytime functional impairments.

All extraction and reasoning steps in the framework were constrained to this predefined ontology. The model was not allowed to generate or infer items outside the ontology, and all identified risk factors and early signals were required to be directly or indirectly supported by the original text.

To improve reproducibility and clinical consistency, we operationally constructed an OSAHS-specific ontology based on established sleep-medicine knowledge, relevant clinical and epidemiological literature, and expert consensus, with reference to concepts and information provided by the American Academy of Sleep Medicine (AASM) and the European Respiratory Society (ERS).

In this ontology, risk factors were defined as pre-existing characteristics or conditions associated with increased susceptibility to OSAHS, whereas early signals were defined as patient- or observer-reported manifestations potentially suggestive of sleep-disordered breathing. The complete ontology definitions, inclusion criteria, and representative examples are provided in Supplementary Tables S1, S2.

Risk factor layer: risk factors were organized into four hierarchical categories: (1) anatomical and physiological factors, referring to body morphology or airway structure associated with upper airway narrowing or collapse tendency, including obesity or weight gain, increased neck circumference or short-neck phenotype, mandibular retrognathia or micrognathia, tonsillar/adenoidal hypertrophy, tongue enlargement, and nasal structural abnormalities or chronic nasal obstruction (e.g., deviated septum, chronic congestion). (2) Sleep-related behaviors, referring to posture and sleep habits that may affect airway patency, including supine sleeping position, insufficient sleep duration, irregular sleep–wake rhythm, alcohol consumption before sleep, and excessive food intake before bedtime. (3) Lifestyle-related factors, referring to long-term habits that may increase OSAHS risk through neuromuscular or airway stability mechanisms, including smoking, chronic alcohol use, physical inactivity, habitual late-night schedules, and high-stress or sedentary lifestyles. (4) Comorbid health conditions, including hypertension, type 2 diabetes, cardiovascular disease, chronic upper airway diseases (e.g., rhinitis, sinusitis), endocrine disorders (e.g., thyroid dysfunction), and long-term psychological stress, anxiety, or depression.

Early signal layer: early signals refer to patient-perceived or observer-reported manifestations associated with sleep-disordered breathing, including habitual or loud snoring, nocturnal choking or perceived apnea, morning dry mouth or sore throat, excessive daytime sleepiness, fatigue, morning headache, attention decline, memory impairment, and related symptoms.

All concepts were mapped to standardized ontology entries. Detailed definitions and example expressions extracted from real narratives are reported in Supplementary Tables S1, S2.

### 2.5 Relevance gate

The first inference step was a relevance gate that determined whether each text contained information potentially related to OSAHS risk. Each text was assigned to one of three relevance levels: strongly relevant, possibly relevant, or not relevant.

Strongly relevant texts explicitly mentioned OSAHS related risk factors or early signals. Possibly relevant texts contained vague, implicit, or incomplete sleep-related cues that required further reasoning. Not relevant texts included content unrelated to sleep disordered breathing or nonspecific sleep complaints without identifiable risk information.

This relevance gate served both as an efficiency mechanism and as a safeguard against inappropriate extraction from irrelevant content.

### 2.6 Internal reasoning strategy

Based on the relevance level, the model applied a structured reasoning strategy under domain knowledge constraints. For strongly relevant texts, the model directly extracted explicitly stated risk factors and early signals from the predefined ontology. For possibly relevant texts, the model performed guided, evidence driven reasoning to surface latent cues that could be associated with risk factors or early signals, while strictly requiring textual evidence for any extraction. For not relevant texts, a conservative relevance check was performed against the ontology. If no association was identified, the text was labeled as containing no risk factors and no early signals. If a potential association was detected, the text was processed following the possibly relevant reasoning pathway.

Across all inference branches, early signals were identified prior to risk factors, reflecting clinical reasoning patterns and supporting interpretability.

### 2.7 Normalization of extracted concepts

To address the linguistic variability inherent in real-world narrative data, a rule-guided normalization strategy integrated with a large language model (LLM) was implemented. Leveraging the LLM's intrinsic capability for semantic abstraction and paraphrase understanding, extracted linguistic expressions were standardized into canonical entries within a predefined clinical ontology. Specifically, the LLM was tasked with identifying semantically equivalent colloquial utterances—despite their syntactic or lexical heterogeneity—and systematically mapping them to uniform clinical concepts. For example, the expressions “I've gained a lot of weight” and “my BMI is too high” were both normalized by the LLM to the core clinical concept of obesity/weight gain. This normalization step, empowered by the LLM's semantic processing capacity, ensured cross-textual consistency of the extracted information, thereby establishing a critical foundation for reliable data aggregation and comparative analysis across different narrative sources.

Concomitantly, a constrained evidence attribution framework was executed by the LLM, which capitalized on its ability to align abstract clinical concepts with their corresponding textual referents. Two strict criteria governed this process: (1) the evidence span must be a contiguous segment of the original narrative text; (2) the span must contain explicit semantic cues that directly support the clinical concept being extracted. To illustrate, given the original narrative “I snore loudly at night and feel extremely sleepy during the day,” the LLM attributed the extracted concept snoring to the contiguous evidence span “snore loudly,” while linking the concept daytime sleepiness to the semantically indicative span “feel extremely sleepy during the day”—a mapping enabled by its inherent capacity to associate semantic meaning with specific textual segments.

### 2.8 Model output and validation

For each text, the framework produced structured outputs including normalized risk factors and early signals, allowing multiple items per text. Binary presence labels were then assigned: the binary variable risk label was assigned a value of 1 when at least one OSAHS-related risk factor was identified and 0 otherwise. The binary variable signal label was assigned a value of 1 when at least one early signal was identified and 0 otherwise. Based on the combination of these labels, each text was categorized into one of four text-level categories: texts containing both risk factors and early signals, texts containing only risk factors, texts containing only early signals, or texts containing neither.

In addition, the framework assigns an overall confidence level (high, medium, or low) to each output for interpretability purposes only. Importantly, this confidence level is not used in determining the final prediction labels or in any downstream decision-making process. The four-class text-level labels (none, ES only, RF only, RF + ES) are determined exclusively based on rule-based categorization according to whether at least one risk factor and/or early signal is identified. The confidence label generated by the model was used only as descriptive metadata for qualitative human inspection and was not incorporated into classification decisions or automated screening logic. Because self-reported confidence from large language models may not correspond to calibrated uncertainty, the confidence output in the present study should not be interpreted as a formally validated uncertainty estimate. Model performance was evaluated using confusion matrices, accuracy, and macro averaged precision, recall, and F1 score, and was compared with multiple baseline text classification models.

## 3 Experiment

The task addressed in this study is the classification of patient-generated free-text descriptions into multiple risk-related categories. Such text is typically informal, heterogeneous, and weakly structured, posing challenges for conventional supervised models that rely on fixed lexical or syntactic patterns (23). To examine whether a pre-trained large language model equipped with knowledge-guided reasoning can serve as a practical solution for public health risk screening, we conducted comparative experiments against supervised neural classifiers. It should be noted that these methods represent different paradigms: OSAHSrisk-LLM performs zero-shot inference without task-specific training, whereas baseline models require full supervised training. Therefore, this comparison aims to illustrate practical applicability under low-resource conditions, rather than to establish a universally optimal model (27, 28).

The proposed OSAHSrisk-LLM, built on GPT-5.1, was evaluated alongside four baseline models: CNN, Text-CNN, Transformer, and BERT. These baselines cover a range of commonly adopted architectures for text classification, including convolution-based approaches and transformer-based language models. All experiments were conducted under the same task definition and evaluation protocol to ensure comparability.

### 3.1 Comparative experimental design

For the baseline models (CNN, Text-CNN, Transformer, and BERT), a supervised learning paradigm was adopted. We employed stratified five-fold cross-validation. In each fold, 80% of the data were used for training and 20% for testing. The reported performance represents the average across the five-fold. This split allowed for an unbiased assessment of each model's generalization ability on unseen data.

Baseline models were trained using standard preprocessing pipelines, including basic text cleaning and Chinese tokenization. CNN and Text-CNN used the jieba tokenizer with randomly initialized 300-dimensional word embeddings and standard convolution-pooling architectures. The transformer baseline adopted SentencePiece-based Chinese subword tokenization with positional encoding, using a lightweight two-layer encoder with four attention heads. The BERT baseline used the official bert-base-chinese pretrained model and its corresponding WordPiece tokenizer. All baseline models used a maximum sequence length of 256 and were optimized using Adam or AdamW with a learning rate of 1e-3, batch size of 32, and a unified early stopping strategy based on validation macro-*F*1. Our intention was to establish representative supervised benchmarks, rather than to perform exhaustive hyperparameter optimization, which would not reflect realistic deployment conditions in many public health settings. All baseline models, including CNN, Text-CNN, Transformer, and BERT, were trained using a stratified five-fold cross-validation strategy. Model optimization was performed using Adam or AdamW, with early stopping based on validation macro-*F*1 for model selection. For each fold, the model checkpoint selected at the final stopping epoch was evaluated on the corresponding held-out fold to obtain test performance. These baseline models were implemented using commonly adopted configurations reported in prior Chinese text-classification studies.

In contrast, OSAHSrisk-LLM does not rely on parameter training on the target dataset. Instead, GPT-5.1 was used as the inference engine within an automated analysis pipeline. All text samples were processed using the same prompts, inference rules, and postprocessing procedures. To support large-scale and consistent evaluation, we implemented an automated script to submit batched requests to the GPT-5.1 interface, collect structured outputs, and compute evaluation metrics. This design ensured that the evaluation of OSAHSrisk-LLM was reproducible and free from manual intervention.

The classification task was formulated as a four-category text-level problem, reflecting different combinations of OSAHS-related risk factors and early signals. Both the proposed framework and baseline models were evaluated under this unified task setting.

### 3.2 Evaluation metrics

Multiple evaluation metrics were used to assess four-class text classification performance, including accuracy, class-wise precision, class-wise recall, class-wise *F*1-score, and macro-averaged precision, recall, and *F*1-score. Accuracy provides an overall indication of the model's predictive effectiveness by measuring the proportion of correctly classified instances. Precision focuses on the reliability of positive predictions, defined as the proportion of true positives among all instances predicted as positive. Recall evaluates the model's ability to capture relevant cases and is calculated as the ratio of correctly identified positives to the total number of actual positive instances. The *F*1-score combines precision and recall into a single metric by computing their harmonic mean, thereby reflecting the trade-off between prediction accuracy and coverage. Collectively, these indicators constitute a comprehensive evaluation framework that enables a more thorough understanding of the model's performance.

## 4 Results and discussion

### 4.1 Results

In this study, each text was assigned to one of four mutually exclusive categories according to the presence of OSAHS-related risk factors and early signals. Specifically, Class 0 represents texts containing neither risk factors nor early signals (None), Class 1 represents texts containing early signals only (ES only), Class 2 represents texts containing risk factors only (RF only), and Class 3 represents texts containing both risk factors and early signals (RF + ES).

Table 1 reports the experimental results of OSAHSrisk-LLM and several baseline models on the four-class text-level classification task. Overall, OSAHSrisk-LLM achieved the highest classification accuracy of 92.9%, significantly outperforming BERT (80.5%), transformer (76.4%), Text-CNN (72.3%), and CNN (68.0%). These results demonstrate the effectiveness of the proposed framework in identifying OSAHS-related risk information from patient-generated text. In addition, we report macro-averaged precision, recall, and *F*1-score, which were computed by averaging the four class-wise values in Table 1, treating all categories equally.

**Table 1:** Experimental results for each model.

Model	Precision (%)				Recall (%)				***F*** **1-score (%)**				Accuracy (%)
	3	2	*1*	*0*	3	2	*1*	*0*	3	2	*1*	*0*	
OSAHSrisk-LLM	94.4	94.5	86.7	84.4	94.4	94.1	87.8	84.4	94.4	94.3	87.2	84.4	92.9
OSAHSrisk-LLM (20%)	92.0	93.1	85.0	82.0	93.5	92.0	86.5	83.0	92.7	92.5	85.7	82.5	91.0
Text–CNN	77.7	79.8	51.4	20.7	83.8	76.5	48.6	18.8	80.6	78.1	50	19.7	72.3
BERT	83.7	86.3	66.2	62.5	80.6	82.4	68.9	93.8	82.1	84.3	67.5	75	80.5
Transformer	83.1	81.1	52.5	32.1	84.3	86.6	41.9	28.1	83.7	83.7	46.6	30	76.4
CNN	73.8	76.5	41.2	8	81	73.9	37.8	6.2	77.3	75.2	39.4	7	68

For comparability, an additional evaluation of OSAHSrisk-LLM was also performed on the corresponding 20% held-out test folds used in the supervised baseline experiments.

Beyond overall accuracy, OSAHSrisk-LLM consistently demonstrated strong performance across all categories, whereas baseline models showed marked variability depending on class type. This pattern suggests that the proposed framework is less sensitive to category imbalance and better suited for real-world screening scenarios.

Specifically, for Class 3 (RF + ES), which contains the most explicit and comprehensive risk-related information, OSAHSrisk-LLM achieved a precision, recall, and *F*1-score of 94.4%, reflecting its strong capability to identify texts that simultaneously include both risk factors and early signals. For Class 2 (RF only), the model maintained similarly high performance, achieving an *F*1-score of 94.3%, indicating reliable recognition of texts that describe OSAHS-related risk factors even in the absence of early signals.

Importantly, OSAHSrisk-LLM also demonstrated robust performance on more challenging categories. For Class 1 (ES only), the model achieved an *F*1-score of 87.2%, suggesting effective detection of early warning signals when explicit risk factors are not present. For Class 0 (None), the model obtained an *F*1-score of 84.4%, showing a strong ability to correctly distinguish non-risk-related texts from those containing subtle or implicit OSAHS-related cues.

In contrast, the baseline models showed pronounced performance degradation on the more difficult categories. Text-CNN and CNN performed poorly on Class 0 (None), with *F*1-scores of only 19.7% and 7.0%, respectively. The Transformer model also struggled on categories lacking explicit risk-related information, particularly Class 1 (ES only) and Class 0 (None). Although BERT achieved high recall for Class 0, OSAHSrisk-LLM showed higher overall accuracy and higher *F*1-scores across the four classes.

Overall, OSAHSrisk-LLM substantially outperformed four representative baseline models in the four-class text-level classification task. The proposed framework achieved the highest overall accuracy and demonstrated more stable class-level performance, especially for minority categories. These results support the overall effectiveness of the complete OSAHSrisk-LLM framework for identifying textual mentions of OSAHS-related risk factors and early signals from real-world patient-generated narratives under the defined four-class classification setting.

### 4.2 Discussion

**Figure 2:**
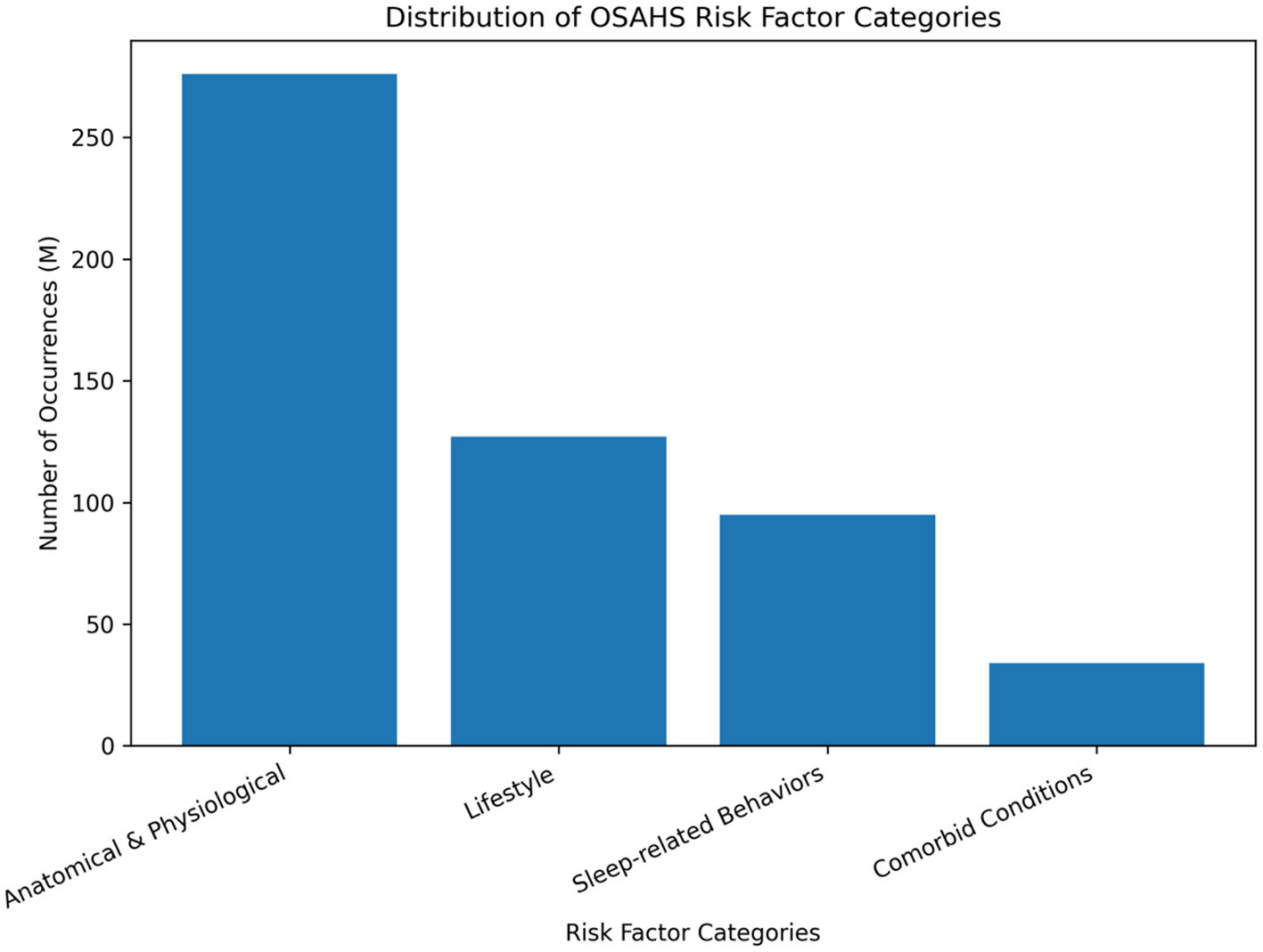
The figure shows the distribution of extracted OSAHS risk factors across four major categories. The *y*-axis represents the total number of occurrences, which exceeds the number of texts due to the presence of multiple risk factors within a single narrative.

**Figure 3:**
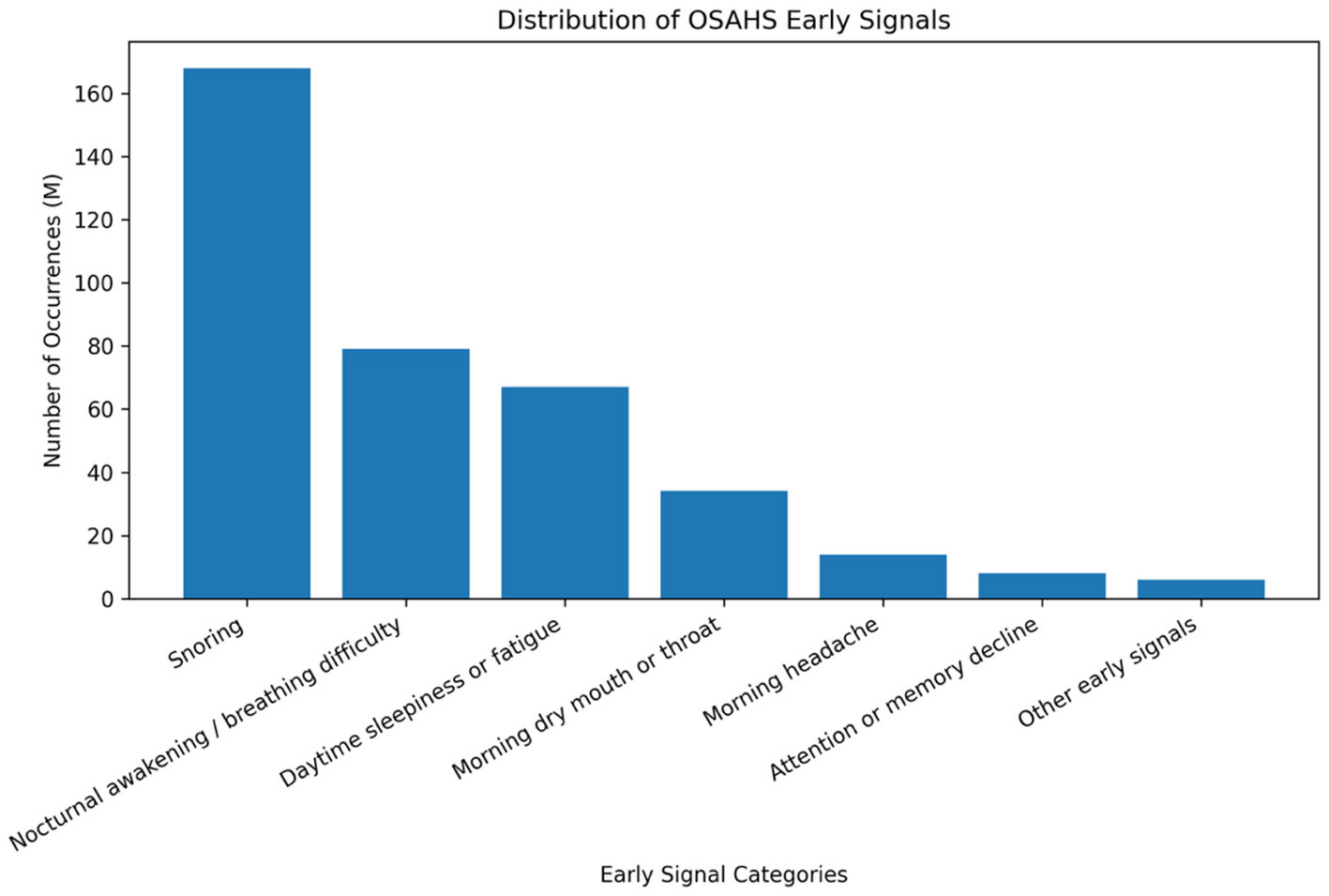
The figure illustrates the frequency of different OSAHS-related early signals identified from patient-generated text. Multiple early signals may co-occur within the same text, resulting in a total occurrence count greater than the number of texts.

Beyond providing context for model evaluation, the overall distribution of risk factors and early signals reflects how OSAHS-related risk is commonly expressed by individuals in nonclinical settings, as shown in Figures 2, 3. Rather than presenting isolated symptoms or single risk attributes, patient-generated narratives often convey a constellation of experiences that jointly signal elevated risk. This pattern is consistent with the multifactorial pathophysiology of OSAHS, in which anatomical predisposition, behavioral factors, and physiological responses interact over time. The frequent co-occurrence observed in this study therefore mirrors the underlying clinical reality, where OSAHS rarely develops from a single factor in isolation (29, 30).

Importantly, this multi-dimensional expression of risk information suggests that early identification efforts should focus on recognizing patterns of combined cues rather than relying on the presence or absence of individual indicators. From this perspective, the distributions shown in Figures 2, 3 not only describe the dataset but also highlight the complexity that any effective screening approach must address when operating on patient-generated text (21).

The distribution of early signals shown in Figure 3 further illustrates how OSAHS-related symptoms are perceived and communicated by patients. The dominance of snoring and nocturnal breathing discomfort reflects the salience of symptoms that are either directly experienced or externally observed, often prompting concern from family members or bed partners. Daytime sleepiness and fatigue, which interfere with daily functioning, also feature prominently, indicating that functional impairment is a key driver of symptom reporting.

In contrast, morning symptoms and cognitive complaints appear less frequently, despite their recognized association with OSAHS in clinical studies. This discrepancy may arise because such manifestations are more gradual, less specific, or less readily attributed to sleep-related breathing problems by patients themselves. The observed distribution therefore highlights a critical gap between clinical knowledge and patient perception, emphasizing the importance of early signal-based approaches that can capture subtle yet meaningful symptom patterns before more severe or long-term consequences become apparent.

Taken together, the distributions of risk factors and early signals reveal that OSAHS-related information in patient-generated text is shaped by both underlying clinical mechanisms and subjective reporting tendencies. These findings underscore the value of analyzing free-text narratives for early risk identification, as they capture experiential and behavioral dimensions that are often absent from structured data (31, 32). In this context, the proposed framework is well positioned to leverage such information by systematically identifying and integrating multiple forms of risk-related evidence within a single narrative (33, 34). We emphasize that early OSAHS risk identification in this study refers to detecting risk factors and early warning signals expressed in social and online health spaces, rather than predicting confirmed disease. The framework operates at a preclinical stage, where individuals may not yet have undergone formal medical evaluation. Its purpose is to support population-level surveillance and risk awareness, not to replace clinical diagnosis. Such an approach aligns with infodemiology and digital public health research, where early signals in public discourse are leveraged to inform health education and early intervention strategies.

The above distributional patterns also provide a direct explanation for the design choices of OSAHSrisk-LLM. As shown in Figure 3, early signals are frequently reported in patient-generated text and often constitute the most salient information available in informal narratives, sometimes appearing in the absence of clearly articulated risk factors (35). This observation supports the decision to explicitly model early signals as a primary target rather than treating them as secondary features. By prioritizing early signal identification and subsequently linking them to risk factors through structured reasoning, OSAHSrisk-LLM aligns with how OSAHS-related risk information is naturally expressed by patients and enables more effective identification of texts that reflect early or preclinical risk states (36).

To further investigate the limitations of the proposed framework, we conducted a qualitative analysis of representative false positive (FP) and false negative (FN) cases. A common FP pattern occurs when users describe general sleep discomfort or transient conditions, such as short-term insomnia caused by stress or acute illness. Although these expressions are semantically similar to OSAHS early signals, they do not reflect chronic or risk-related symptoms, leading the model to incorrectly classify general sleep complaints as potential warning signs. Another typical FP scenario involves figurative or exaggerated language (e.g., “I feel like I stop breathing when I'm extremely tired”), which activates risk-related keywords without sufficient clinical context, thereby triggering erroneous predictions.

FN cases mainly arise from highly implicit expressions, where users describe symptoms indirectly rather than using canonical clinical terms. For example, phrases such as “I always wake up feeling unrefreshed” implicitly indicate daytime sleepiness but may not be recognized by the model. Such linguistic variability increases the difficulty of accurate extraction. Additionally, some FN errors occur in long narratives where multiple weak cues are scattered across sentences. Although each cue is individually insufficient to trigger detection, their joint presence is clinically meaningful, which the current model may fail to aggregate effectively.

Several limitations of this study should be noted. First, the dataset was collected from Chinese online health platforms, and the findings may not be directly generalizable to other languages or populations. Second, although expert consensus labeling was applied, patient-generated text is inherently subjective and ambiguous, which may introduce residual uncertainty in the reference labels (37). Previous studies have also highlighted the challenges of uncertainty quantification and confidence calibration in medical large language models, including work by Madrid et al. evaluating uncertainty-aware ChatGPT-based assessment in medical examination settings (38). Finally, the proposed framework is designed for early risk identification rather than clinical diagnosis, and its outputs should be interpreted as supportive screening information rather than definitive clinical conclusions.

## 5 Conclusion

In this study, we investigated the early identification of obstructive sleep apnea hypopnea syndrome risk factors and early signals from patient-generated free text using a large language model–based framework. By analyzing real-world narratives collected from online health platforms, we demonstrated that OSAHS-related risk information is commonly expressed in a multi-dimensional and experience-oriented manner, with early signals often serving as the most salient and accessible cues. To address these characteristics, we proposed OSAHSrisk-LLM, which integrates relevance screening, structured reasoning, and clinical knowledge constraints to systematically extract and organize risk factors and early signals from unstructured text.

Experimental results showed that the proposed framework outperformed conventional text classification models in a four-class text-level classification task, particularly under highly imbalanced data conditions. More importantly, the design of OSAHSrisk-LLM allows early signals and risk factors to be explicitly modeled and linked within a single narrative, improving both robustness and interpretability. These advantages suggest that the framework may be useful for exploratory public health surveillance and preliminary identification of OSAHS-related textual signals. Prospective validation in clinically characterized populations is required before deployment in real-world screening workflows. Overall, this work highlights the potential of combining large language models with structured reasoning to support early OSAHS risk identification and provides a practical foundation for extending text-based screening approaches to other sleep-related or chronic conditions.

## Data Availability

The dataset used in this study consists of publicly available user-generated online health narratives collected from online health consultation platforms. Because the raw texts may contain sensitive contextual information, the full corpus is not publicly released. De-identified representative examples, ontology definitions, annotation guidelines, and supplementary methodological details are provided in the article and Supplementary Materials. Additional requests regarding processed research materials may be directed to the corresponding author and will be considered under appropriate privacy and ethical constraints.
