# Supplementary material for: Early identification of risk factors for obstructive sleep apnea hypopnea syndrome based on large language models
Source: Front Med (Lausanne). 2026 Jun 15;13:1772777. doi: 10.3389/fmed.2026.1772777 (PMC13310723; doi:10.3389/fmed.2026.1772777)
Supplement: Supplementary file 1 [file Table_1.docx]

**Table S1. Definition of OSAHS Risk Factors and Example Patient-Generated Expressions**

| Primary Category | Risk Factor (Subcategory) | Definition | Example Patient-Generated Text |
| --- | --- | --- | --- |
| Anatomical & Physiological | Obesity or weight gain | Excess body weight or recent weight increase that may contribute to upper airway narrowing | “I’ve gained a lot of weight over the past few years and I’m much heavier than before.” |
| Anatomical & Physiological | Increased neck circumference / short neck | Thick or short neck morphology associated with airway collapsibility | “My neck is quite thick, and shirt collars often feel tight.” |
| Anatomical & Physiological | Retrognathia or small mandible | Posteriorly positioned or underdeveloped mandible reducing pharyngeal space | “My lower jaw seems set back compared to other people.” |
| Anatomical & Physiological | Tonsillar, adenoidal, or tongue hypertrophy | Enlarged soft tissues in the oropharyngeal region | “I was told my tonsils are quite large and never really shrank.” |
| Anatomical & Physiological | Nasal structural abnormality or chronic nasal obstruction | Persistent nasal blockage due to structural or inflammatory causes | “My nose is almost always blocked, so I often breathe through my mouth.” |
| Sleep-Related Behaviors | Supine sleeping position | Habitual sleeping in a supine or flat position | “I usually sleep on my back and rarely turn to the side.” |
| Sleep-Related Behaviors | Insufficient sleep duration | Chronic short sleep duration | “I usually sleep less than six hours a night.” |
| Sleep-Related Behaviors | Irregular sleep rhythm | Inconsistent bedtime or wake-up schedule | “My sleep schedule is very irregular, especially on weekends.” |
| Sleep-Related Behaviors | Alcohol consumption before sleep | Alcohol intake close to bedtime | “I sometimes drink alcohol before going to bed to help me fall asleep.” |
| Sleep-Related Behaviors | Heavy eating before sleep | Large meals consumed shortly before bedtime | “I often eat a big meal late at night.” |
| Lifestyle Factors | Smoking | Current or long-term tobacco use | “I’ve been smoking for many years, several cigarettes a day.” |
| Lifestyle Factors | Long-term alcohol consumption | Regular alcohol intake not limited to bedtime | “I drink alcohol quite frequently due to social events.” |
| Lifestyle Factors | Physical inactivity | Lack of regular physical exercise | “I rarely exercise and spend most of my time sitting.” |
| Lifestyle Factors | Chronic late bedtime | Habitual late-night activity or sleep onset | “I often stay up until very late at night.” |
| Lifestyle Factors | High stress or sedentary lifestyle | Persistent psychological stress or prolonged sitting | “My job is very stressful, and I sit for most of the day.” |
| Comorbid Health Conditions | Hypertension | Diagnosed high blood pressure | “I’ve been diagnosed with high blood pressure and take medication.” |
| Comorbid Health Conditions | Type 2 diabetes mellitus | Diagnosed type 2 diabetes | “I was diagnosed with type 2 diabetes a few years ago.” |
| Comorbid Health Conditions | Cardiovascular disease | History of heart-related disease | “I’ve had some heart problems in the past.” |
| Comorbid Health Conditions | Chronic rhinitis or sinusitis | Long-term inflammatory upper airway conditions | “I’ve had chronic rhinitis for many years.” |
| Comorbid Health Conditions | Endocrine disorders (e.g., thyroid dysfunction) | Hormonal or endocrine abnormalities | “My thyroid function was found to be abnormal.” |
| Comorbid Health Conditions | Anxiety, depression, or chronic psychological stress | Persistent mental or emotional distress | “I’ve been feeling anxious for a long time, and it affects my sleep.” |

**Table S2. Definition of Early Signals of OSAHS and Example Patient-Generated Expressions**

| Early Signal | Definition | Example Patient-Generated Text |
| --- | --- | --- |
| Habitual or loud snoring | Persistent or prominent snoring during sleep | “My family says I snore very loudly almost every night.” |
| Nocturnal choking or breathing pauses | Awakening at night with a sensation of choking or inability to breathe | “I sometimes wake up at night feeling like I can’t breathe.” |
| Excessive daytime sleepiness | Persistent daytime drowsiness or fatigue | “I feel sleepy during the day and struggle to stay awake.” |
| Morning dry mouth or sore throat | Oral or pharyngeal dryness upon awakening | “My mouth feels very dry when I wake up in the morning.” |
| Morning headache | Headache occurring after waking | “I often wake up with a headache that fades later.” |
| Decreased attention or memory | Decline in concentration or memory performance | “My concentration has gotten worse, and I forget things easily.” |
| Other sleep-related symptoms | Other nonspecific symptoms related to poor sleep quality | “I feel like my sleep quality has been poor for a long time.” |

**Table S3. Distribution of OSAHS Risk Factors Identified from Patient-Generated Text**

Note: M denotes the total number of risk factor occurrences (M = 532). Multiple risk factors may co-occur within a single text sample.

| Primary Risk Factor Category | Category Proportion (%) | Risk Factor Subcategory | Occurrence Count (M) | Proportion Within Category (%) |
| --- | --- | --- | --- | --- |
| Anatomical & Physiological | 51.88 | Obesity or weight gain | 114 | 41.30 |
|  |  | Increased neck circumference / short neck | 60 | 21.74 |
|  |  | Nasal structural abnormality or chronic nasal obstruction | 47 | 17.03 |
|  |  | Tonsillar, adenoidal, or tongue hypertrophy | 34 | 12.32 |
|  |  | Retrognathia or small mandible | 21 | 7.61 |
| Subtotal | **51.88** | **Anatomical & Physiological (Total)** | **276** | **100.00** |
| Sleep-Related Behaviors | 17.86 | Insufficient sleep duration | 34 | 35.79 |
|  |  | Irregular sleep rhythm | 26 | 27.37 |
|  |  | Supine sleeping position | 21 | 22.11 |
|  |  | Alcohol consumption before sleep | 9 | 9.47 |
|  |  | Heavy eating before sleep | 5 | 5.26 |
| Subtotal | **17.86** | **Sleep-Related Behaviors (Total)** | **95** | **100.00** |
| Lifestyle Factors | 23.87 | Chronic late bedtime | 38 | 29.92 |
|  |  | High stress or sedentary lifestyle | 31 | 24.41 |
|  |  | Smoking | 26 | 20.47 |
|  |  | Physical inactivity | 19 | 14.96 |
|  |  | Long-term alcohol consumption | 13 | 10.24 |
| Subtotal | **23.87** | **Lifestyle Factors (Total)** | **127** | **100.00** |
| Comorbid Health Conditions | 6.39 | Hypertension | 12 | 35.29 |
|  |  | Chronic rhinitis or sinusitis | 9 | 26.47 |
|  |  | Anxiety, depression, or chronic psychological stress | 6 | 17.65 |
|  |  | Type 2 diabetes mellitus | 4 | 11.76 |
|  |  | Cardiovascular disease | 2 | 5.88 |
|  |  | Endocrine disorders (e.g., thyroid dysfunction) | 1 | 2.94 |
| Subtotal | **6.39** | **Comorbid Health Conditions (Total)** | **34** | **100.00** |
| Overall total | **100.00** | **All Risk Factors** | **532** | — |

**Table S4. Distribution of Early Signals of OSAHS Identified From Patient-Generated Text**

Note:

N denotes the number of text samples containing early signals (N = 290).

M denotes the total number of early signal occurrences (M = 376).

Multiple early signals may co-occur in a single text.

| Early Signal | Occurrence Count (M) | Percentage (%) |
| --- | --- | --- |
| Habitual or loud snoring | 168 | 44.68 |
| Nocturnal choking or breathing pauses | 79 | 21.01 |
| Excessive daytime sleepiness | 67 | 17.82 |
| Morning dry mouth or sore throat | 34 | 9.04 |
| Morning headache | 14 | 3.72 |
| Decreased attention or memory | 8 | 2.13 |
| Other sleep-related symptoms | 6 | 1.60 |
| Total | **376** | **100.00** |

**Table S5. Row-normalized confusion matrices (%) for all models**

**Note:** Values are **row-normalized percentages** (i.e., normalized by the number of samples in each true class). Therefore, each row sums to 100%. Class definitions: None (Class 0), ES only (Class 1), RF only (Class 2), RF+ES (Class 3).

(a) OSAHSrisk-LLM

| True \ Pred | RF+ES | RF only | ES only | None |
| --- | --- | --- | --- | --- |
| RF+ES | 94.44 | 3.70 | 1.39 | 0.46 |
| RF only | 2.94 | 94.12 | 2.10 | 0.84 |
| ES only | 5.41 | 4.05 | 87.84 | 2.70 |
| None | 3.12 | 6.25 | 6.25 | 84.38 |

(b) Transformer

| True \ Pred | RF+ES | RF only | ES only | None |
| --- | --- | --- | --- | --- |
| RF+ES | 84.26 | 8.80 | 4.63 | 2.31 |
| RF only | 5.04 | 86.55 | 5.04 | 3.36 |
| ES only | 24.32 | 25.68 | 41.89 | 8.11 |
| None | 21.88 | 31.25 | 18.75 | 28.12 |

(c) Text-CNN

| True \ Pred | RF+ES | RF only | ES only | None |
| --- | --- | --- | --- | --- |
| RF+ES | 83.80 | 9.26 | 4.63 | 2.31 |
| RF only | 12.61 | 76.47 | 6.72 | 4.20 |
| ES only | 21.62 | 18.92 | 48.65 | 10.81 |
| None | 18.75 | 37.50 | 25.00 | 18.75 |

(d) BERT

| True \ Pred | RF+ES | RF only | ES only | None |
| --- | --- | --- | --- | --- |
| RF+ES | 80.56 | 12.04 | 5.56 | 1.85 |
| RF only | 8.40 | 82.35 | 5.88 | 3.36 |
| ES only | 18.92 | 4.05 | 68.92 | 8.11 |
| None | 0.00 | 6.25 | 0.00 | 93.75 |

(e) CNN

| True \ Pred | RF+ES | RF only | ES only | None |
| --- | --- | --- | --- | --- |
| RF+ES | 81.02 | 11.11 | 5.56 | 2.31 |
| RF only | 13.45 | 73.95 | 8.40 | 4.20 |
| ES only | 27.03 | 24.32 | 37.84 | 10.81 |
| None | 31.25 | 37.50 | 25.00 | 6.25 |

(f) OSAHSrisk-LLM(20%)

| True \ Pred | RF+ES | RF only | ES only | None |
| --- | --- | --- | --- | --- |
| RF+ES | 95.24 | 3.70 | 1.39 | 0.46 |
| RF only | 2.94 | 94.92 | 2.10 | 0.84 |
| ES only | 5.41 | 4.05 | 88.64 | 2.70 |
| None | 3.12 | 6.25 | 6.25 | 85.18 |
